# Screening and Molecular Analysis of Single Circulating Tumor Cells Using Micromagnet Array

**DOI:** 10.1038/srep16047

**Published:** 2015-11-05

**Authors:** Yu-Yen Huang, Peng Chen, Chun-Hsien Wu, Kazunori Hoshino, Konstantin Sokolov, Nancy Lane, Huaying Liu, Michael Huebschman, Eugene Frenkel, John X. J. Zhang

**Affiliations:** 1 Thayer School of Engineering, Dartmouth College, Hanover, NH 03755; 2 Department of Biomedical Engineering, The University of Texas at Austin, Austin, TX 78712-0238; 3 Department of Biomedical Engineering, University of Connecticut, Storrs, CT 06269-3247; 4 Department of Imaging Physics, Division of Diagnostic Imaging, The University of Texas MD Anderson Cancer Center, Houston, TX 77030; 5 Harold C. Simons Comprehensive Center of the University of Texas Southwestern Medical Center, Dallas TX 75390.

## Abstract

Immunomagnetic assay has been developed to detect rare circulating tumor cells (CTCs), which shows clinical significance in cancer diagnosis and prognosis. The generation and fine-tuning of the magnetic field play essential roles in such assay toward effective single-cell-based analyses of target cells. However, the current assay has a limited range of field gradient, potentially leading to aggregation of cells and nanoparticles. Consequently, quenching of the fluorescence signal and mechanical damage to the cells may occur, which lower the system sensitivity and specificity. We develop a micromagnet-integrated microfluidic system for enhanced CTC detection. The ferromagnetic micromagnets, after being magnetized, generate localized magnetic field up to 8-fold stronger than that without the micromagnets, and strengthen the interactions between CTCs and the magnetic field. The system is demonstrated with four cancer cell lines with over 97% capture rate, as well as with clinical samples from breast, prostate, lung, and colorectal cancer patients. The system captures target CTCs from patient blood samples on a standard glass slide that can be examined using the fluorescence *in-situ* hybridization method for the single-cell profiling. All cells showed clear hybridization signals, indicating the efficacy of the compact system in providing retrievable cells for molecular studies.

Detection and enrichment of target cells, such as stem cells^1^, disseminated tumor cells (DTCs)^2^, and circulating tumor cells (CTCs)^3^ from heterogeneous suspensions play a central role in biomedical research and clinical practice. In particular, circulating tumor cells (CTCs) have been shown to closely relate to cancer metastasis^4^,^5^ providing information to assist cancer studies. First, accurate enumeration of CTCs can be used as a key indicator for cancer diagnosis, prognosis, and cancer treatment monitoring^6^. Beyond enumeration, advanced single cell characterization techniques, such as fluorescence *in-situ* hybridization (FISH)^7^, reverse transcription polymerase chain reaction (RT-PCR)^8^, and quantitative RT-PCR^9^, can provide insights into the biologic characterization of the CTCs. CTCs have the potential of providing a non-invasive “liquid-biopsy” to study the heterogeneity of cancer cells and eventually aid the development of personalized therapy^10^,^11^,^12^. A combination of rapid enumeration and molecular profiling are critical to exploit the full potential of CTCs.

The challenges associated with CTC detection and analyses begin with the natural scarcity of CTCs (the estimated ratio between CTCs and normal leukocytes is 1:10^7^–10^9^), therefore platforms for CTC detection with high sensitivity, specificity, and reliability are in need^4^. A great number of separation systems have been developed, such as an antibody mediated immunoassay^13^, size-based filtration method^14^, fluorescence-activated cell sorting (FACS)^15^, immunomagnetic separation^16^,^17^,^18^,^19^, and dielectrophoresis force separation^20^, and others as summarized in previous reviews^21^. Among the popular methods, the immunomagnetic cell separation assay, which works by selectively labeling the CTCs with magnetic nanoparticles, and using an external magnetic field to capture target cells, provides an effective solution for the translational clinical applications^22^,^23^,^24^. The immunomagnetic assay exhibits good sensitivity and specificity that arises from the cancer specific antibody-antigen interactions. In addition, the large effective range of magnetic attraction enables the larger channel size and allows for higher throughput. The immunomagnetic assay can also be integrated with multiple separation mechanisms, such as size filtration and inertial focusing^25^. The immunomagnetic assay has been widely applied for cell separation from heterogeneous suspensions^16^.

Approaches with engineered functional surface using techniques such as chemically modified three dimensional micro/nano-structures are proposed to enhance the sensitivity of rare cell detection^26^,^27^,^28^,^29^. For immunomagnetic assays, several isolation methods integrated with non-functionalized 3-D structures in the microchannel have been employed for particle sorting and cell detection with large populations^30^,^31^,^32^. To achieve high detection sensitivity and retain both the physical and biological integrities of the target cells, we propose a patterned thin-film micromagnet design, which can be integrated into a microchip based immunomagnetic assay to improve the detection and analysis of the CTCs.

## Results

### Design and fabrication of micromagnet-integrated microfluidic screening system

**Figure 1:**
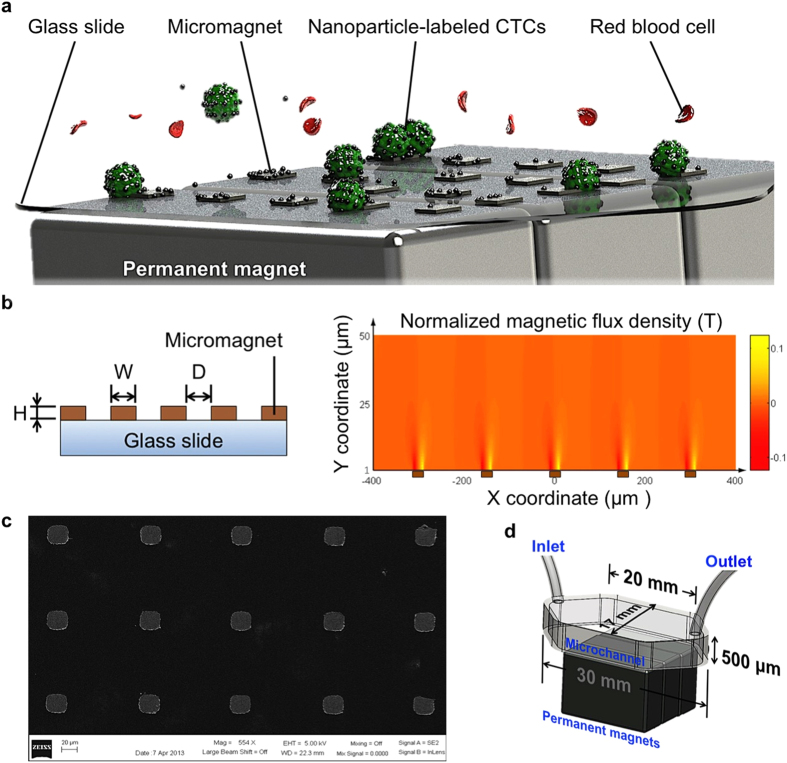
Micromagnet-integrated microfluidic device and characterization of micromagnets. (**a**) Schematic illustration of the glass substrate patterned with micromagnets for immunomagnetic isolation of cancer cells. Permanent magnets provide external magnetic field and magnetize micromagnets to induce local magnetic field enhancement. Magnetic nanoparticles-labeled CTCs are captured to the channel substrate by the arrays of micromagnets as the blood sample flows through the microchannel. (**b**) Schematic of the patterned thin-film micromagnet array showing the lateral dimensions, thickness, and spatial periodicity. Magnetic field distribution of an array of five micromagnets (brown blocks) within a microfluidic channel space obtained using COMSOL. The strongest spots of magnetic field are around the edges of the micromagnets. (**c**) SEM image of fabricated micromagnets. (**d**) Schematic shows the setup of the screening system with dimensions of the microchannel.

When placed in an external magnetic field, the micromagnets can be magnetized to generate a localized strong magnetic field that can enhance the attractive interactions between cells and the capture surface in the microchannel. Compared to the conventional magnetic activated cell sorting system, where permanent magnets are used as the only magnetic flux source, the micromagnet approach increases the magnetic trap density throughout the whole microchannel surface and local magnetic field gradient. The micromagnets are designed to yield better capture sensitivity, achieve better capture distribution, and facilitate the downstream analyses. To fulfill these purposes, several design factors need to be considered, including the thickness, the lateral dimension, and the spatial periodicity of the micromagnets. Thickness of a micromagnet determines the magnitude of the magnetic force and the vertical effective range of the micromagnet. To minimize the physical damages to the cells due to collision, we decreased the thickness of the micromagnets compared with previous structures. Lateral dimension determines the lateral magnetic effective range of each micromagnet. Another key design parameter is the spatial periodicity of the micromagnet array, which plays an important role in altering the distribution of the captured cells. Figure 1a shows the principle of the micromagnet implementation.

We simulated the magnetic field distribution with the presence of an array of five micromagnets linearly aligned on the substrate. A two-dimensional model was built with FEM simulation software (COMSOL Multiphysics). Lateral dimensions, thickness, and spatial periodicity are represented as W, H, and D, respectively (Fig. 1b). The normalized magnetic flux density above the micromagnets (1 μm ~ 50 μm) is plotted (Fig. 1b). We examined the local magnetic field on the surface of the micromagnets, and found that thin-film nickel micromagnets increased the magnetic field 8-fold maximum in the vicinity of the micromagnet edge (SI, Fig. 1). Each micromagnet was in a cubic shape with the dimensions of 20 μm × 20 μm × 250 nm (W × W × H) so that the length of each micromagnet was designed to be compatible with the diameters of CTCs, which range from 12 μm to 25 μm^33^. Thickness of a micromagnet at the nanoscale largely reduces the risk of damaging the CTCs during the capture. According to our previous theoretical endeavor of the micromagnet approach^34^, the periodicity plays an important role in tuning the surface magnetic field to distribute the captured CTCs, and facilitate the subsequent fluorescence imaging and identification steps. We choose a 100 μm spatial periodicity (D) based on the calculated effective range of magnetic field created by a single micromagnet (30 μm) and the size of cancer cell (20 μm). Detailed theoretical derivation has been described in the previous report^34^. A standard thermal deposition technique was employed to fabricate the micromagnets. A scanning electron microscope (SEM) picture shows arrays of fabricated micromagnets (Fig. 1c).

For experimental demonstration, arrayed ferromagnetic Ni thin-film is deposited on the substrate of the microchannel. Figure 1d illustrates the integrated micromagnets immunomagnetic CTC detection system. A polydimethylsiloxane (PDMS) chip is bonded to a standard glass slide integrated with micromagnets forming a hexagonal micro-chamber with a height of 500 μm, width of 17 mm, and length of 30 mm. Three permanent magnets (Block NdFeB magnet, product of 42 MGOe, grade N42, 3/4″ × 1/2″ × 7/32″) are placed outside the microfluidic device with alternate polarities. The sample is introduced to the microchannel by a syringe pump. CTCs are labeled with magnetic nanoparticles (Ferrofluid^TM^, Veridex, LLC, NJ) based on cancer specific anti-epithelial cell adhesion molecule (anti-EpCAM) expression. When the sample is flowed through the microchannel, nanoparticles-labeled CTCs are magnetically captured on the channel substrate, while normal hematocytes, such as red blood cells (RBCs) and leukocytes, flow out of the microchannel. After the screening, the captured CTCs, now on the standard glass slide, can be immunofluorescently stained for imaging, identification, and further molecular studies.

The proposed approach combines the magnetic forces at different length-scales so that permanent magnets provide a long-range magnetic attraction, and the micromagnets provide the short-range retaining force on the target cells. The layout of the micromagnet is designed to be an array format offering distributed magnetic traps that help overcome the limitations of the cell and nanoparticle aggregation that may affect the morphological and fluorescent signals of the target cells.

### Experimental results of the micromagnet-integrated microfluidic screening system

**Figure 2:**
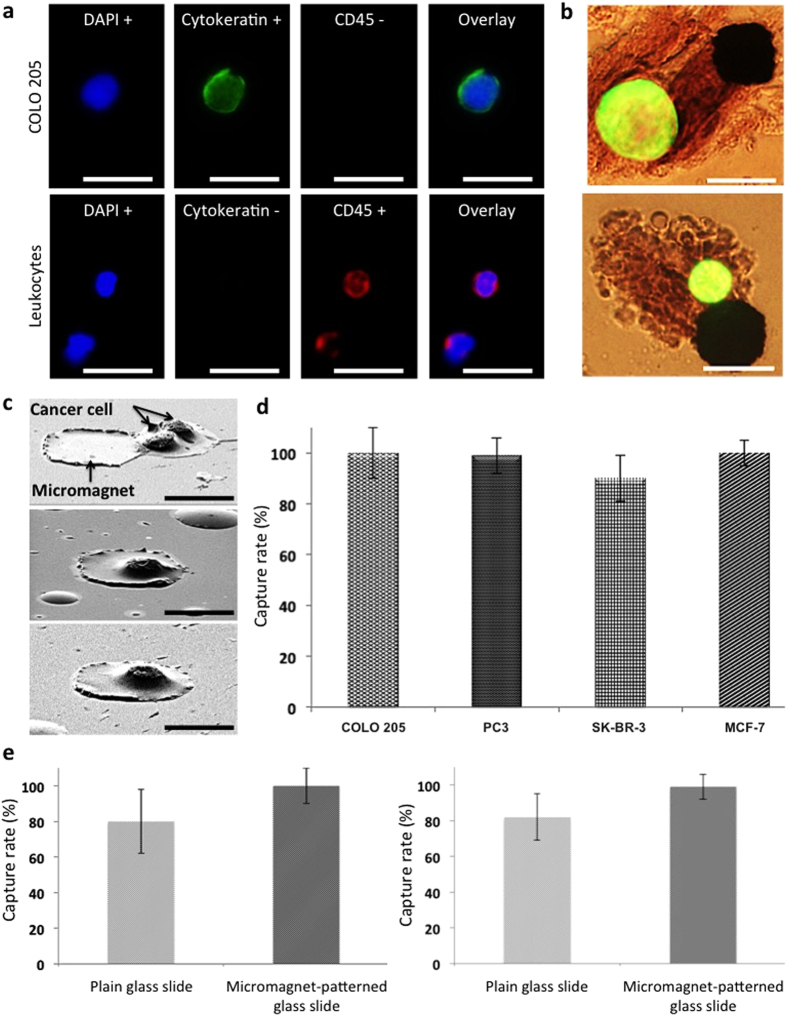
Characterization of micromagnets-integrated microchip by the comparative spiked experiments. (**a**) Fluorescence images of a COLO 205 cell and two leukocytes (COLO 205: DAPI+, Cytokeratin+, and CD45−; Leukocyte: DAPI+, Cytokeratin−, and CD45+). Scale bar is 20 μm. (**b**) Overlay of fluorescence and bright field images of FITC-stained COLO 205 cells (green) captured by micromagnets. Scale bar is 20 μm. (**c**) SEM images of a doublet of COLO 205 cells captured next to a micromagnet and a COLO205 captured on top of a micromagnet. Scale bar is 20 μm. (**d**) Capture rates of screenings spiked with COLO 205, PC3, SK-BR-3, and MCF-7 cell lines. The average capture rate with micromagnet-patterned glass slides was 97.3%. (**e**) Comparative screening showed that micromagnet-patterned glass slide showed higher capture rate and stability than the plain glass slide. Scale bar is 20 μm.

To examine the efficacy of the micromagnet integrated assay, four different human cancer cell lines were spiked into normal human blood: including COLO 205 (colorectal), SK-BR-3 and MCF-7 (breast), and PC3 (prostate). Capture rate is defined as the ratio of the number of cancer cells captured in the screened samples to the average number of cancer cells prepared on multiple control slides. Typically, two control slides are prepared from the same cell suspension at the same time as the blood sample is spiked. The number of spiked CTCs is obtained in the following way: when the screening blood sample is spiked with cancer cells, equal amount of the same cell suspension is dropped on glass slide as control samples. The average number of cancer cells found in the control samples indicates the number of cancer cells spiked into the screening blood samples, and is used to calculate the capture rate. To identify cancer cells, experimental slides were immunofluorescently stained with DAPI (blue), CK-FITC (green), and CD45-Alexa Fluor 594 (red). Cancer cells exhibit DAPI+, CK+, and CD45−, while the main interfering leukocytes are DAPI+, CK−, and CD45+. One sample cancer cell and two leukocytes are shown in Fig. 2a. The cells are identified based on the morphological characteristics and fluorescent signals by a trained observer, and each cell is further confirmed by multiple physicians. This combinational approach has been used for CTC studies, and has been proved to be able to effectively differentiate WBCs and CTCs^13^,^35^. In order to further verify the feasibility of our identification process in a systematic and objective manner, image analysis of captured cells has been made to conduct computer-aided cell identification^18^. The result ensured its agreement with the cell identification by the observer and physicians. Since the micromagnets increase the surface retaining force in a short range, we can directly observe micromagnet capturing cancer cells with a strong fluorescent signal and an intact shape (Fig. 2b). SEM images show cancer cells being captured by micromagnets (Fig. 2c). Negative control testing with *CellSearch*^*TM*^ system has been conducted by multiple groups^36^,^37^. Based on these early studies, we have indeed performed comparable screening tests where blood samples from both the cancer patients and the healthy donors were used. The operator of the apparatus did not have the information about the donors. Through the six trials, no CTC was found from healthy donors’ blood samples. Figure 2d shows the capture rates of all four spiked cancer cell lines using the micromagnet-patterned glass slide (COLO 205: 99.5 ± 9.9%, n = 15; PC3: 99.1 ± 7.3%, n = 8; SK-BR-3: 90.4 ± 9.3%, n = 5; and MCF-7: 100 ± 4.8%, n = 5). We performed comparative spiked experiments using plain glass slides (without micromagnets) with two cell lines, COLO 205 and PC3. The capture rates were 79.7 ± 17.7% for COLO 205 (n = 15) and 82.1 ± 12.9% for PC3 (n = 8) (Fig. 2e). The implementation of the thin-film micromagnets optimized the assay in the following ways: First, an average 18.4% increase in capture rate was achieved (COLO 205: 19.8% improvement, *p* = 0.0012; PC3: 17% improvement, *p *= 0.006). Secondly, the micromagnets reduced the variations of the capture rates (COLO 205: ±7.8%; PC3: ±5.6%), indicating improved working stability and thirdly, the micromagnets demonstrated the versatility of the assay by the screening of four different cancer cell lines.

**Figure 3:**
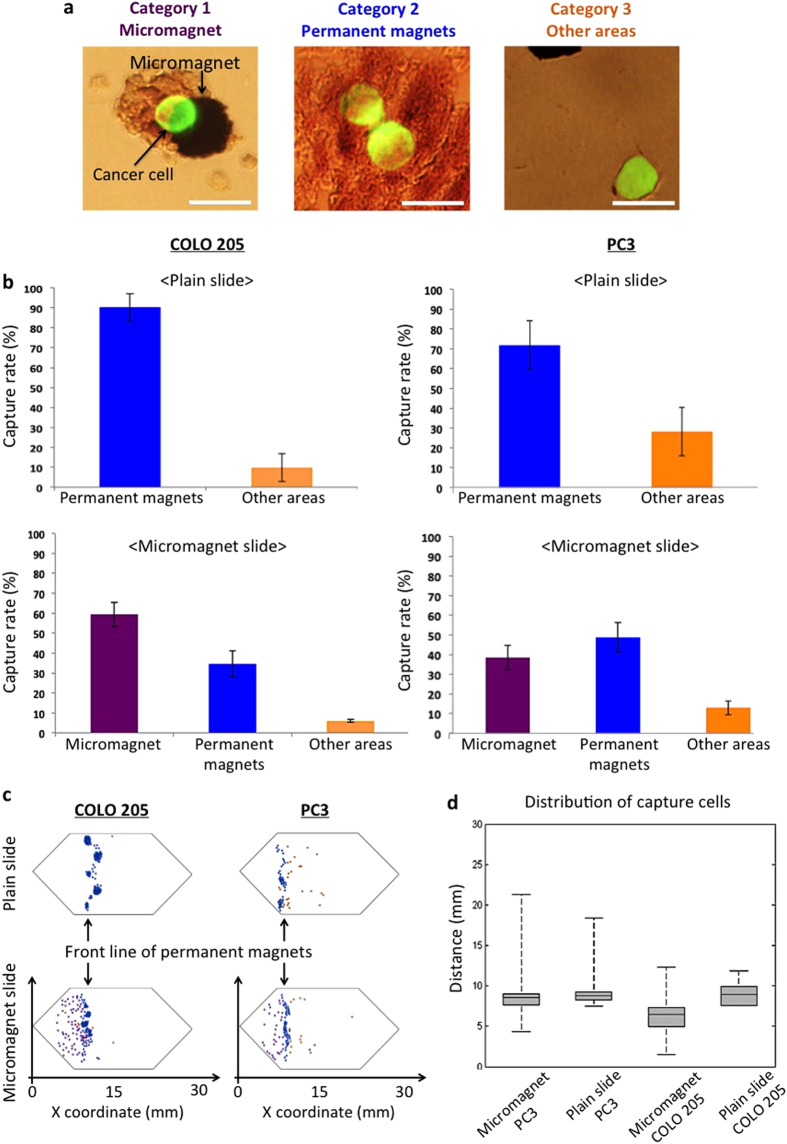
Characterization of micromagnet-integrated microchip with three categories of captured cancer cells. (**a**) Overlay of fluorescence images and bright field images of FITC-stained COLO 205 cells (green). Category 1 of captured cancer cells showing that a cancer cell is captured by a micromagnet. Category 2: cells are captured by permanent magnets. Category 3: cells are captured on other areas. Scale bar is 20 μm. (**b**) Capture rates of COLO 205 and PC3 cells for three categories with/without micromagnets. For plain glass slide, most COLO 205 cells were captured on the front line of permanent magnets. In addition, patterned micromagnets reduced the capture rate of COLO 205 captured elsewhere. (**c**) Mapping of COLO 205 and PC3 locations on plain and micromagnet slides. (**d**) Distribution analysis of COLO 205 and PC3 recorded on the plain and micromagnet slides.

On the experimental slides, we observed the CTCs being captured by the micromagnets, where there were also magnetic nanoparticles aggregating around the micromagnets (Fig. 3a). The aggregated magnetic nanoparticles increase the effective range of the micromagnets. The morphology clearly showed the interactions between the CTCs and micromagnets. To investigate the impact of the micromagnets, the captured CTCs were categorized into three groups based on how they were captured (Fig. 3a), 1) by micromagnets; 2) by permanent magnets; 3) other area. Specifically, CTCs captured by permanent magnets refer to those target cells found near the front edge of the permanent magnets, but not attached to micromagnets. Figure 3b shows the percentages of each category for the plain and micromagnet slides respectively. For the plain glass slide, most COLO 205 cells (90.2% of spiked cells) were captured by permanent magnets, while 9.8% of cells were captured otherwise. For the micromagnet-patterned glass slide, micromagnets attracted 59.4% of spiked cancer cells during the screening process, while 34.6% of spiked COLO 205 cells were still captured by the permanent magnets. Only 6% of spiked cells were captured elsewhere. We could observe similar phenomenon in the spiked experiment with PC3 – about 38.5% of spiked PC3 cells were magnetically attracted by the micromagnets. The number of CTCs captured by permanent magnets dropped from 71.8% to 48.7% with the micromagnets. The increased ratio of cells captured by the micromagnets demonstrates the significant role of the micromagnets, verifying that the increase of the capture rates can be attributed to the implementation of the thin-film micromagnets.

After the screening experiments, we recorded the position of each captured cancer cell to study the distribution patterns. COLO 205 and PC3 were used as model cells, and the location maps of captured CTCs on micromagnet and plain slides of COLO 205 and PC3, respectively (Fig. 3c). We then measured the x coordinate of each cell using the left end of the microchannel as the origin, and summarized the distribution patterns for the four experimental conditions (Fig. 3d). On each box, the central mark is the median value, the edges of the box are the 25^th^ and 75^th^ percentiles, and the whiskers extend to the most extreme data points. In all the four cases, the median values were around 9 mm, which was the location of the front edge of the permanent magnets array. It indicates that the permanent magnets provide the major attractive forces. However, on micromagnet slides, 25% of the cells were captured before the front edge of the permanent magnets, while 0% (no cell) was captured before this edge on plain slides. Besides, the total ranges of cell distribution area increased 55% for PC3 (from 11 mm to 17 mm), 175% for COLO 205 (from 4 mm to 11 mm). The dramatic increase of this distribution range can be explained since the micromagnets create multiple additional potential magnetic trapping sites that are capable of capturing the target cells in a distributed format to prevent cell damage by stacked nanoparticles. The patterned thin-film micromagnets implementation provides an effective way to alleviate the cell aggregation issues of the assay since that more cells are captured by micromagnets rather than the areas with an aggregation of nanoparticles.

Comparing the results between the two cell lines, we found that PC3 cells tended to migrate further than COLO 205 cells, under the same experimental conditions. On micromagnet slides, the median x coordinate of captured CTCs was 9 mm for PC3 and 6 mm for COLO 205, while the maximum value was 21 mm for PC3, and 12 mm for COLO 205. On average, PC3 traveled 1.6 times further than COLO 205. This spatial difference between the two cell lines could be attributed to the expression levels of the surface biomarker. PC3 cells, which have lower EpCAM expression^24^, conjugate with a smaller number of magnetic nanoparticles than COLO 205 cells. As such, a PC3 cell is subjected to smaller magnetic attractive forces, thus can move longer horizontal distance before being captured than a COLO 205. This cell-dependent distance information can be used reversely as a fundamental clue for cell type identification. Our system, for the first time, provides spatial distinguishable information that can potentially be used for cancer cell phenotyping based on the biomarker expression levels.

### Downstream analysis of captured circulating tumor cells from the spiked screenings

**Figure 4:**
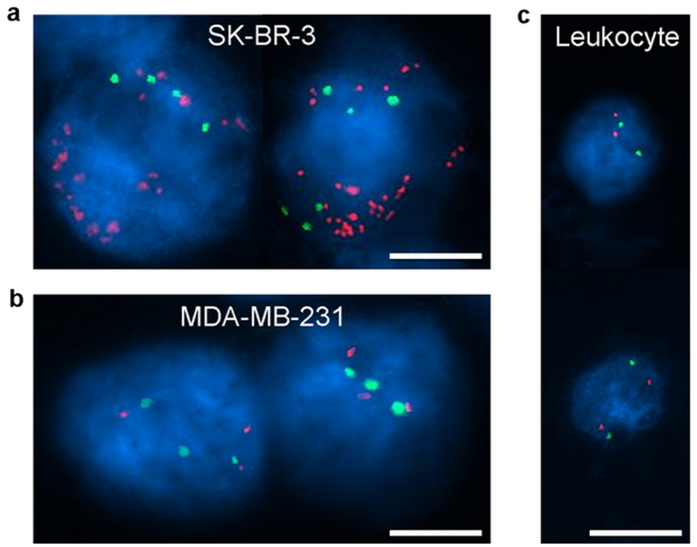
FISH results of SK-BR-3 cells, MDA-MB-231 cells, and control cells (leukocytes) in the spiked experiments. (**a**) All cells showed clear hybridization signals for HER-2 in orange and CEP17 in green. SK-BR-3 cells showed 20 and 30 copies of HER-2, while 4 and 6 copies of CEP17 were measured. (**b**) MDA-MB-231 cells showed 3 copies of HER-2 and 3 copies of CEP17. (**c**) Leukocytes showed 2 copies of HER-2 and 2 copies of CEP17. Scale bar is 10 μm.

Amplification of HER-2 (human epidermal growth factor receptor 2, HER-2/ERBB2) identifies the cell as clearly a breast cancer cell and is seen progression of breast cancer. Copy number of HER-2 is largely associated with a shorter survival and an increased risk of re-occurrence of cancer^38^,^39^. To demonstrate that our system provides retrievable CTCs for molecular profiling at single cell scale, we used the FISH method to evaluate the copy number of HER-2 and the ratio number of HER-2 to chromosome (CEP17) of the captured breast cancer cells (SK-BR-3 and MDA-MB-231) in spiked experiments. We performed fluorescence in-situ hybridization (FISH) on the captured CTCs to study the HER-2 expression. Figure 4 shows the experimental results of FISH analysis showing the hybridization signals for HER-2 (orange) and CEP17 (green) of HER-2 positive cancer cell line (SK-BR-3), HER-2 negative cancer cell line (MDA-MB-231), and leukocytes as control cells. SK-BR-3 cells show a high level of HER-2 amplification, with more than 30 copies (Fig. 4a). While, the MDA-MB-231 cells show no significant amplification of HER-2, only with 3 copies (Fig. 4b). As a negative control, we studied leukocyte, and it showed negative HER-2 amplification (Fig. 4c). The average ratio number of SK-BR-3 is 5.4 showing that SK-BR-3 is a HER-2 positive cell line, and the MDA-MB-231 cell shows the ratio number to be 1. The control cell, which is a leukocyte here, shows the ratio number to be 1. Observation of SK-BR-3 (HER-2 positive) and MDA-MB-231 (HER-2 negative) cells here accords well with the previous conclusions^40^,^41^. It approves the efficacy of our system in providing retrievable cells for molecular studies.

### Screening and single cell profiling of clinical samples from cancer patients

We applied the developed micromagnet-integrated system in screening blood samples from the late stage cancer patients, and successfully found cancer cells from the clinical samples with four different types of metastatic cancers - colorectal, lung, prostate, and breast cancers. All patients had not received any therapeutic treatments before the donation of blood samples for the screening experiments. Table 1 summarizes the pathological information and CTC screening experimental results of the patient samples. Notably, for samples 3 and 4, we performed comparative experiments using the micromagnet-integrated screening system and the “gold-standard” *CellSearch*^*TM*^ system (Veridex, LLC, NJ). In both cases, our device outperformed the *CellSearch*^*TM*^ system that no cell was found in the *CellSearch*^*TM*^ system.

**Figure 5:**
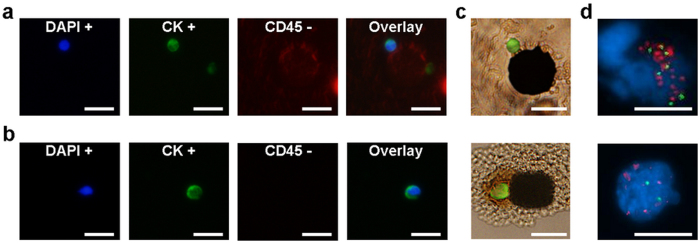
Cancer patient study results. (**a,b**) Fluorescence signals of the cancer cells found from breast cancer patients #9 and #10 from the Table 1. (**c**) Direct capture of CTCs onto the micromagnet was observed. (**d**) FISH results of the cells found from cancer patients #9 and #10. All cells showed clear hybridization signals for HER-2 in orange and CEP17 in green. CTCs captured from patient blood samples showed amplification of HER-2. Scale bar is 20 μm.

The CTCs found from cancer patients were identified using the same immune-staining method (Fig. 5a,b). Similarly, we observed the direct capture of CTCs by the micromagnets (Fig. 5c). We categorized the captured CTCs in the same way, and found that 54% of the CTCs were captured by micromagnets, 28% were captured by permanent magnets, and 19% were captured elsewhere. The high ratio of the CTCs captured by the micromagnets was consistent with the results of the spiked experiments. It again confirms the significance of the micromagnets in facilitating the capture of the CTCs.

FISH analysis has been performed for CTCs captured from patient samples #9 and #10. Large copy numbers of HER-2 (sample #9: more than 16 copies; sample #10: 6 copies) were found in CTCs from cancer patient samples (Fig. 5d). The ratio numbers of all three CTCs were larger than 2.2 (sample #9: larger than 2.2; sample #10: 3) suggesting that the captured CTCs were HER-2 positive cancer cells.

## Discussion

This screening system combined the advantages of the immunomagnetic assay and micromagnet-integrated microfluidic device providing the capability of isolating CTCs from whole blood samples with high capture efficiency, high sensitivity, high throughput, high versatility, and simple experimental setup. Incorporation of micromagnets patterned in the microchannel enhanced the retaining magnetic force locally, reduced the extent of aggregated free nanoparticles and cell aggregation by efficiently utilizing the channel space, increased the reliability of achieving high capture efficiency with less variation, and achieved single-cell resolution. The system has been used to screen four cell lines from three different cancer types showing high capture efficiency. Furthermore, the screening system has been successfully applied to clinical screening of blood samples from patients with cancer. We also performed single-cell fluorescence *in-situ* hybridization (FISH) analysis to measure the copy numbers and ratio numbers of HER-2 and CEP17 of SK-BR-3 (HER-2 positive), MDA-MB-231 (HER-2 negative), and CTCs from a cancer patient sample. The local gradient tuning micromagnet activate cell separation system provides a versatile tool for potential single cell based recognition and monitoring of cancer.

## Methods

### Screening system

The microchannel was made by a standard molding technique using polydimethylsiloxane (PDMS). Surfaces of PDMS chips and glass slides were treated with O_2_ plasma followed by a bonding process to form microchannel. One end of the microchannel device was connected to a reservoir, while the other end was connected to a waste collection tube. A syringe pump was used to draw the blood sample from the reservoir through the microchannel, and collected the waste liquid at the tube. An automatic rotational microfluidic device holder was developed to rotate the orientation of microfluidic device during the screening process, including the separation step and the flushing step. The screening system provided the function of rocking the reservoir to mix the blood sample while screening. Six samples could be screened at the same time to increase the screening throughput. More details can be found in^17^,^18^. Based on the mathematical model we built in the previous publication^42^, the microchannel was inversely placed during the separation process to achieve high capture efficiency.

### Fabrication of micromagnets

Arrayed micromagnets were defined by the lift-off technique. A photoresist (AZ5209) was spun on a glass slide and patterned by the standard photolithography process. A 15 nm thick chromium layer was thermally deposited as an adhesion layer. Next, a thin-film of nickel layer (250 nm thick) was thermally deposited. Photoresist was removed to form the nickel micromagnets.

### Blood sample collection

All blood samples were obtained from healthy donors and cancer patients in the UT Southwestern Medical Center at Dallas with informed consent from all participants under an IRB-approved protocol. The project was approved by the Institutional Biosafety Committee (IBC) and the Advisory Committee on Human Research at University of Texas at Austin. All screenings were performed in accordance with the declaration of Helsinki.

### Blood sample preparation

Blood was collected in a CellSave tube (Veridex, Janssen Diagnostic LLC, NJ) and centrifuged at 800 G for 10 minutes. Supernatant containing plasma was replaced with dilution buffer solution (Veridex, Janssen Diagnostic LLC, NJ) for the total volume to be 3.5 mL. Suspensions of magnetic nanoparticles (Fe_3_O_4_ magnetic nanoparticle, Ferrofluid^TM^, Veridex, Janssen Diagnostic LLC, NJ) functionalized with anti-epithelial cell adhesion molecules (anti-EpCAM) and capture enhancement reagent were added to the blood sample tube and then incubated in a strong magnetic field for 10 minutes before the screening experiment. The flow rate was kept at 2.5 mL/hr during the screening process. Magnetic force was used to magnetically trap the nanoparticle-labeled cancer cells on the channel substrate. Phosphate buffered saline (PBS) was used to flush the microchannel after the screening process. More details can be found in^17^,^18^.

### Cell Culture

MCF-7, PC3, SK-BR-3, and COLO 205 cells were used as cancer cell models to demonstrate molecular-specific cellular imaging. Cells were cultured in RPMI-1640 (1X, Gibco, Grand Island, NY) medium containing 20% fetal bovine serum (FBS, Gibco, Grand Island, NY), and harvested at ~90% confluence with trypsin. Trypan blue was used to stain the cells, and haemocytometer was used to count the cells in the suspension. Cell suspensions containing ~3 × 10^5^ cells/mL were re-suspended in complete media. Meanwhile, a diluted cell suspension containing ~3 × 10^4^ cells/mL (equivalent to 200 cells/10 μL) were prepared as the spiked screen sample.

### Immunofluorescence staining and imaging

Captured cancer cells were fixed in ice-cold acetone on the channel substrate for 10 minutes. The experimental glass slide was rinsed with 1X PBS and 0.1% Tween-20 (Sigma-Aldrich, St. Louis, MO). The 300 μL blocking buffer (Boca Science Inc., Boca Raton, FL) was added on the sample slide followed by the incubation with 37^o^C for 60 minutes. The cells were then immunofluorescently stained with anti-cytokeratin (mouse anti-cytokeratin, positive test, pan-FITC, Sigma-Aldrich, St. Louis, MO) and anti-CD45 (AlexaFluor 594, negative test, Invitrogen, Grand Island, NY) in staining solution (1X PBS, 0.1% Tween-20, and 1% BSA). Incubation time is 45 minutes with 37^o^C. The slide was immersed in 1X PBS and 0.1% Tween-20 for 5 minutes for three times. Next, the cells were stained with DAPI (stains DNA found in cell nucleus, positive test, Vectashield Mounting Medium with DAPI, Vector Laboratories, Inc., Burlingame, CA). The sample slide was stored in 4^o^C refrigerator for 30 minutes before the observation and cell identification with the inverted microscope (IX81, Olympus, Center Valley, PA).

### Definition of the capture rate

Capture rate is defined in the following way:

We defined the term “capture rate” to quantify the yield of our system. It is calculated based on the ratio between the number of captured cancer cells and spiked cancer cells.


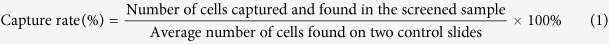


Two control slides were prepared from 10 μL of cell suspension (about 200 cells) when the blood sample was spiked with same volume of cell suspension. Capture rate could be higher than 100% when the number of cells spiked in the blood sample is more than the average number of cells on the two control slides because of the concentration variations in such a small cell suspensions (10 μL).

### Statistical analysis

Data of capture rates are reported as mean ± standard deviation of the mean. If groups had a standard normal distribution and homogenous variances, the group means were compared by an independent t-test, and the variances were compared using a Levene’s test. Differences in the comparative experiments were considered significant with the confidence level at 95% (p < 0.05).

### Fluorescence *in-situ* hybridization (FISH) analysis

After the screening process, the immunofluorescence staining, and the cell identification, the experimental slide was used for fluorescence *in-situ* hybridization analysis. The cover slip was removed by immersing the experimental slide in the 1X PBS solution for two hours. Next, target cancer cells captured and fixed on the glass slide were then studied by the FISH method. LSI HER-2 DNA probe and chromosome 17 centromere (CEP17) DNA probe were used for copy number measurement of HER-2 and CEP17 respectively. A 190 Kb Spectrum Orange directly labeled fluorescent DNA probe specific for HER-2 gene locus. A 5.4 Kb Spectrum Green directly labeled fluorescent DNA probe specific for the alpha satellite DNA sequence at the centromere region of chromosome 17.

## Additional Information

**How to cite this article**: Huang, Y.-Y. *et al.* Screening and Molecular Analysis of Single Circulating Tumor Cells Using Micromagnet Array. *Sci. Rep.* **5**, 16047; doi: 10.1038/srep16047 (2015).

## Supplementary Material

Supplementary Information

Supplementary Figure 1

## Figures and Tables

**Table 1:** Results of screened patient samples with micromagnet-patterned glass slide (pathology information included).

Sample number	Cancer Type	Gender	Ferrofluid/blood (μL/mL)	Flow rate (μL/hr)	Screening volume (mL)	Number of CTCs	Number of CTCs/7.5 mL of blood found from *CellSearch*^*TM*^
1	Colon cancer	M	7.5	2.5	5.0	1	N/A
2	Lung cancer	F	7.5	2.5	10.0	1	N/A
3	Prostate cancer	M	7.5	2.5	7.5	13	0
4	Breast cancer	F	7.5	2.5	7.5	6	0
5	Breast cancer	F	7.5	2.5	7.5	3	N/A
6	Breast cancer	F	7.5	2.5	5.0	10	N/A
7	Breast cancer	F	7.5	2.5	7.5	2	N/A
8	Breast cancer	F	7.5	2.5	7.5	1	N/A
9	Breast cancer	F	7.5	2.5	10.0	22	N/A
10	Breast cancer	F	7.5	2.5	5.0	215	N/A
11	Breast cancer	F	7.5	2.5	7.5	2	N/A
12	Breast cancer	F	7.5	2.5	7.5	6	N/A
13	Breast cancer	F	7.5	2.5	5.0	7	N/A

Comparative experiments were performed for samples 3 and 4 using the micromagnet-integrated screening system and *CellSearch*^*TM*^ system. No cell was found from the *CellSearch*^*TM*^ while CTCs were found in the micromagnet-integrated system.
