# Supplementary material for: Screening and Molecular Analysis of Single Circulating Tumor Cells Using Micromagnet Array
Source: Sci Rep. 2015 Nov 5;5:16047. doi: 10.1038/srep16047 (PMC4633592; doi:10.1038/srep16047)
Supplement: Supplementary Information [file srep16047-s1.pdf]

Screening and Molecular Analysis of Single Circulating Tumor Cells Using Micromagnet Array

*Yu-Yen Huang[1], *Peng Chen[2], Chun-Hsien Wu[2], Kazunori Hoshino[3], Konstantin Sokolov[4], Nancy Lane[5], Huaying Liu[5], Michael Huebschman[5], Eugene Frenkel[5], and John X.J. Zhang[1]

[1] Thayer School of Engineering, Dartmouth College, Hanover, NH 03755
[2] Department of Biomedical Engineering, The University of Texas at Austin, Austin, TX 78712-0238
[3] Department of Biomedical Engineering, University of Connecticut, Storrs, CT 06269-3247
[4] Department of Imaging Physics, Division of Diagnostic Imaging, The University of Texas MD Anderson Cancer Center, Houston, TX 77030
[5] Harold C. Simons Comprehensive Center of the University of Texas Southwestern Medical Center, Dallas TX 75390
* The authors contributed equally to this work
Contact: john.zhang@dartmouth.edu

Numerical simulation methods. Magnetic field simulation was performed using finite element method (FEM) software COMSOL. Two-dimensional model was built with identical size to the real device. Dimensions of a single micromagnet element were 20 μm × 250 nm. The distance between adjacent micromagnets was 150 μm. An array consisting of five micromagnet elements linearly aligned on the substrate was used for demonstration. The physics module of the static magnetic field was chosen in COMSOL, in which the magnetic field was calculated based on relative magnetic permeability of the materials $B=\mu_0 \bullet \mu_r H$. The material of the micromagnet was specified to Nickel, with relative magnetic permeability μ_r=200. The rest of the parts of the model were set to be air (μ_0=1). Given the large dimension ratio of our micromagnet design, magnetic anisotropy dominated the magnetization inside the micromagnets. We set the boundary condition with an external magnetic field (B=0.1 T) along the horizontal direction. This value was set to match the magnetic field found on the surface of the microchannel. After the numerical simulation, the magnetic field data was exported and re-plotted using MATLAB (Natick, MA). **SI, Fig. 1** shows the simulated results of an array of five micromagnets (represented in black blocks) that magnetic field of the vicinity of the micromagnets are 8-fold enhanced.

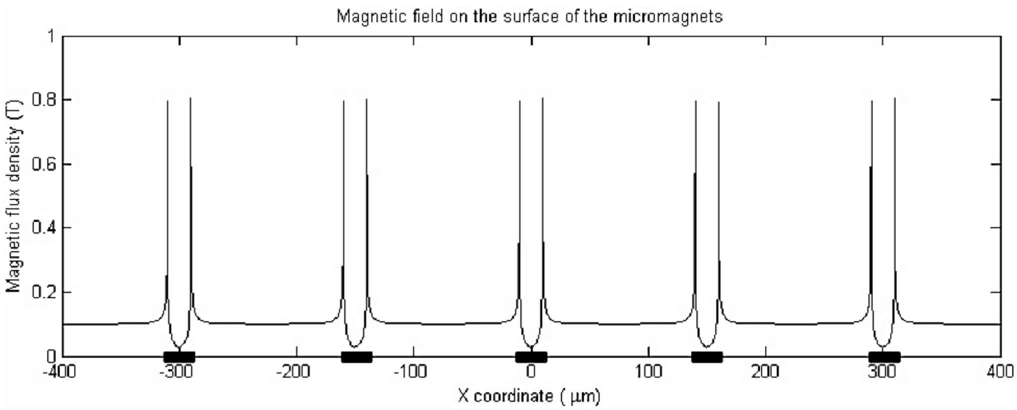

SI, Figure 1 | Normalized magnetic field on the surface of the micromagnets.
