# Supplementary figures and images for: Screening and Molecular Analysis of Single Circulating Tumor Cells Using Micromagnet Array
Source: Sci Rep. 2015 Nov 5;5:16047. doi: 10.1038/srep16047 (PMC4633592; doi:10.1038/srep16047)

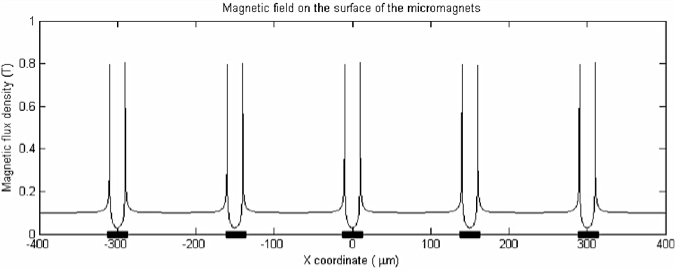

Supplement: Supplementary Figure 1 [file srep16047-s2.jpg]
